# A Case of Severe Abomasal Sand Impaction in a Farmed White-Tailed Deer (*Odocoileus virginianus*) in Florida

**DOI:** 10.3390/ani14111602

**Published:** 2024-05-29

**Authors:** Alireza Rahmani Shahraki, João H. J. Bittar, Samantha M. Wisely, Juan M. Campos-Krauer

**Affiliations:** 1 Department of Large Animal Clinical Sciences, College of Veterinary Medicine, University of Florida, Gainesville, FL 32611, USA; alirezarahmanish@ufl.edu (A.R.S.); jbittar@ufl.edu (J.H.J.B.); 2 Department of Wildlife Ecology and Conservation, University of Florida, Gainesville, FL 32611, USA; wisely@ufl.edu; 3 Emerging Pathogens Institute, University of Florida, Gainesville, FL 32611, USA

**Keywords:** deer, abomasal impaction, sand, Florida

## Abstract

**Simple Summary:**

This case report presents a chronic abomasal sand impaction in a white-tailed deer in Florida. Abomasal impaction in ruminants can result from a low-quality diet or abnormal eating behavior. The farmer reported significant weight loss, abdominal enlargement, and general weakness in the deer. Post-mortem examination revealed a low body condition score, dehydration, and abdominal enlargement. Internal inspection identified pneumonia lesions in the left cranial lung lobe, which microbiological tests attributed to *Trueperella pyogenes*. Notable ruminal bloating was observed, characterized by gas accumulation. The abomasum was palpably firm and enlarged, predominantly filled with sand, except for a small ventral portion, with an estimated sand weight of approximately 5 kg. Although chronic sand impaction was suggested as the cause of death, it is posited that chronic sand-induced damage to the abomasal mucosal layer led to active subacute abomasitis, which compromised the animal’s immune system and predisposed it to secondary infections.

**Abstract:**

The University of Florida’s Cervidae Health Research Initiative (CHeRI) conducted a post-mortem examination of a two-year-old white-tailed doe deceased at a northern Florida white-tailed deer farm. The carcass of the deer had notable emaciation and bloating. Upon opening of the carcass, there was pneumonia and the rumen was tympanic and enlarged. Additionally, the abomasum was distended and contained approximately 5 kg of sand. It is not uncommon for white-tailed deer to engage in geophagia (eating soil or sand), which typically does not result in diseases or fatalities. However, in this animal, we suspect a chronic process that created a physical barrier, hindering nutrient absorption and resulting in physical irritation of the abomasal mucosa with subsequent inflammation. This may have caused a disturbance in immune system function, allowing opportunistic bacteria to colonize and invade other organs, such as the lungs, contributing to the animal’s death.

## 1. Introduction

Abomasal impaction is characterized by the accumulation of compacted ingesta within the abomasum, resulting in distention and enlargement of the organ. This condition leads to decreased abomasal motility, and reduced rumen fluid flow and ingesta movement to the intestine, often accompanied by failure in the aboral transport mechanism [1,2]. Abomasal impaction can be a primary disorder that leads to the development of secondary conditions such as peritonitis [2]. The failure to pass fluid and ingesta to the intestine can result in dehydration, acid–base imbalances, and varying degrees of electrolyte imbalance, including metabolic alkalosis, hypochloremia, and hypokalemia. Weakness and decreased food intake are common consequences of abomasal disorders in farm animals such as deer. In white-tailed deer, geophagia, the consumption of soil, is a relatively common behavior [3]. Geophagia involving natural mineral licks has been documented across North America [3,4,5,6,7,8,9,10,11]. These studies have consistently identified sodium (Na) as the most abundant mineral element present at these ungulate licks, alongside other essential minerals like sulfur (S), calcium (Ca), and magnesium (Mg) [3,5,6,12]. Furthermore, it is normal for young fawns to consume soil and for adults to engage in geophagia to acquire specific minerals and introduce beneficial bacteria into their gastrointestinal tract. In the vast majority of cases, geophagia does not lead to diseases or death [2]. Sand impaction is reported in various animal species, such as cattle, horses, and even elephants (*Elephas maximus*) [13,14]. Sand impaction can occur indirectly through consumption of contaminated food and water with sand [13]. Clinical signs of this progressive disease in different animal species include depression, anorexia, diarrhea, and scant feces [2]. Owners often report abdominal distention, loss of body weight, and weakness to rise. Initially, body temperature is usually normal, but due to negative energy balance, it may become subnormal after several days. Varying degrees of colic with mucosal damage are observed, often followed by bacterial infection, progressive endotoxemia, and ultimately death [15,16]. The aim of this manuscript is to report, for the first time, the occurrence of abomasal sand impaction in white-tailed deer (*Odocoileus virginianus*) in Florida. 

## 2. Case History

A deer farmer in northern Florida reached out to the University of Florida’s Cervidae Health Research Initiative (CHeRI) to request a post-mortem examination of a two-year-old white-tailed doe that was discovered deceased on the morning of 29 December 2017. The deer was stored in a walk-in refrigerator over 12 h until the necropsy was performed the following morning. Prior to its demise, the animal had a history of an enlarged abdomen for over four months and distinctive digestive noises, akin to sloshing, when it moved in its enclosure. Remarkably, these noises and the enlarged rumen did not seem to have a significant impact on the animal’s overall health or reproductive success, aside from a notable weight loss. Four weeks before its demise, the doe underwent a successful laparoscopic artificial insemination, and it did not display any signs of illness until its death.

## 3. Necropsy and Lab Findings

The field necropsy procedures encompassed a thorough examination of the carcass. Upon external examination, the animal was notably lean and had lost body condition, and exhibited bloating. We found no external indications of trauma, wounds, parasites, or any abnormalities. The fur was dry and no secretions in the nose or mouth were observed. There were no signs of anemia, characterized by pale mucosal membranes in the oral cavity and eye. Additionally, the external reproductive organs appeared normal with no secretions. 

**Figure 1:**
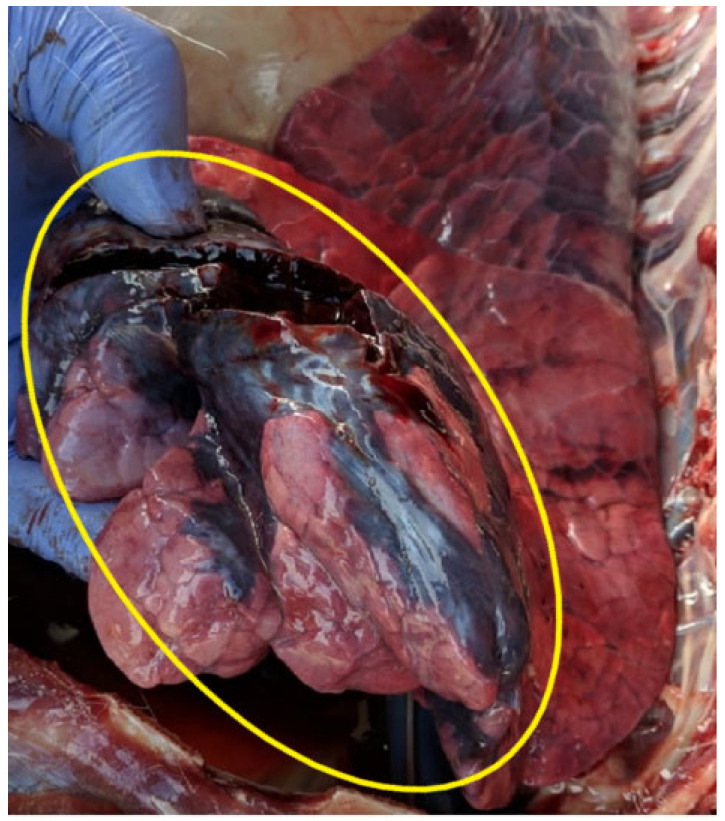
The yellow circle shows the lung lesion caused by *Trueperella pyogenes* in the white-tailed deer (*Odocoileus virginianus*) with severe abomasal sand impaction.

Necropsy of the thoracic cavity revealed lung congestion, consolidation, and compartmentalization, with areas of blood and pus, particularly in the left cranial lung lobe, which exhibited a significant necrotic region measuring 5 to 6 cm (Figure 1). Samples from this area were collected for microbiology testing. Moreover, the heart was normal in size but showed signs of hyperemia and congestion. In the abdominal cavity the spleen was contracted, measuring only 11 cm in diameter and 1 cm in thickness throughout most of the organ. The kidneys were congested and all major organs appeared dehydrated, particularly liver and spleen.

**Figure 2:**
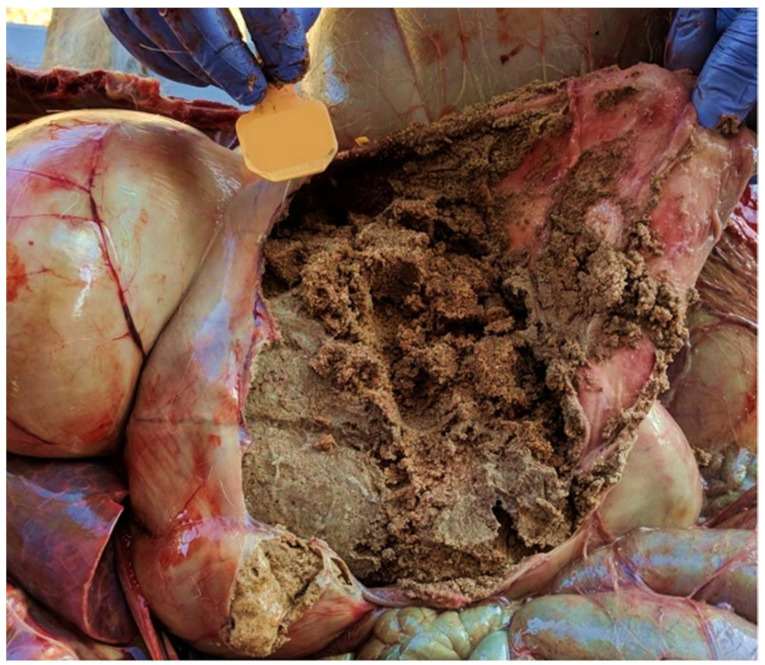
The open abomasum of a female white-tailed deer (*Odocoileus virginianus*). The deer suffered from chronic abomasal sand impaction. The ear tag included in the image as a reference is 5.08 cm by 4.12 cm.

The rumen exhibited marked bloating and contained a substantial amount of gas. The abomasum was enlarged and firm on palpation. Upon incising it was impacted with firm, clear sand, except for a small ventral area that contained a mixture of liquid food and sand on the red mucosa with minimal mucus (Figure 2). These findings indicated a chronic case of abomasal sand impaction. The sand, estimated to weigh 5 kg, exerted dependent pressure on the rumen, causing it to be displaced forward and potentially exerting significant pressure on the diaphragm.

The remaining portions of the gastrointestinal tract were normal. However, the small and large intestines were empty, containing only dry and hard fecal pellets in the rectum. No internal parasites were identified. All reproductive organs were normal and the doe was pregnant, with a 4 × 1.5 cm 20–25 days gestational fetus. 

Samples of vital organs (lungs, heart, liver, kidney, spleen), blood, and fecal pellets were collected and stored at −80 °C freezer for subsequent laboratory analysis. Moreover, two nasal swabs and one rectal swab were obtained and stored in sterile 1.5 mL tubes and universal transport media (UTM) for further testing.

In accordance with the suspected disease, samples were submitted the University of Florida (UF) Microbiology, Parasitology, and Serology Diagnostic Laboratory of the College of Veterinary Medicine. Blood samples were also sent to the Cervidae Health Research Initiative Molecular Ecology Lab at the University of Florida to be tested for epizootic hemorrhagic disease virus (EHDV), bluetongue virus (BTV), West Nile virus (WNV), and Eastern equine encephalitis (EEE) by RT-PCR [17,18,19], and the test results were negative.

Microbiology recovered heavy growth in the lungs of *Trueperella pyogenes*, a bacterium known to cause lesions and septicemia in white-tailed deer. 

## 4. Discussion

Sand impaction is a chronic disease reported in domestic and wild animals [13,20]. The consumption of soil is a common behavior in white-tailed deer whose exact cause remains uncertain [21]. The ingestion of small amounts of soil is frequently observed in post-mortem examinations of calves and lambs [22]. This case represents the first report of severe abomasal sand impaction in an adult white-tailed deer in Florida. While chronic sand impaction was suggested as the cause of death, we believe that chronic subacute abomasitis may have contributed to immune suppression and predisposed the animal to secondary infections. This hypothesis aligns with existing literature [23]. *Trueperella pyogenes* is an opportunistic bacterium commonly found on mucosal surfaces, the rumen wall, and the upper respiratory system of deer and other farm animals [24,25]. Additionally, there is an association between intracranial abscesses in deer and the presence of *Trueperella pyogenes* [26]. In this case, we hypothesize that consistent inflammation in the abomasal wall, leading to an insufficient immune response, contributed to the lung lesions caused by *Trueperella pyogenes*. Reduced abomasal contractions and distended abomasum occurs in animals afflicted with abomasal impaction. Accumulation of sand in the abomasum can impact abomasal motility and contribute to hypomotility and eventually atony. This functional motility disorder can cause distention due to gas accumulation in the forestomach, especially in the rumen [13,27]. In this case, bloat was indicated in the owner’s history and necropsy findings. The lesions in this doe were consistent with prior case reports [8,27]. More severe sand impaction can lead to partial or complete obstruction of the upper gastrointestinal tract, contributing to abdominal pain, decreased appetite, and scant feces [13,28]. The empty intestine in this case is consistent with other previous reports in the literature [1,27]. Additionally, severe dehydration and presumptive electrolyte imbalances occurred due to partial or complete obstruction in the upper alimentary tract, disrupting the natural passage of fluids from the abomasum into the duodenum. Previous case studies reported association between the occurrence of abomasal impaction and biochemical alteration in ruminants [29,30]. In this doe, post-mortem inspection revealed that the small intestines were empty, and the lack of chyme maybe attributed to the presence of hardened sand, which impeded the movement of food. Animals with abomasum obstruction due to sand or ingested feed often feature congestion and hyperemia in the serous layer of the organ [1,20]. In this case, petechiae and ecchymoses were observed on the serous layer of the impacted abomasum. The physical abrasion pressure exerted by sand likely induced mechanical damage to the abomasal wall, resulting in hemorrhage and inflammation, leading to alteration in blood vessel permeability. Furthermore, damage to the serosal layer of the abomasum and alterations in blood vessel permeability facilitate the entry of gastrointestinal microflora, particularly gram-negative bacteria, into the bloodstream. The lipopolysaccharide (LPS) of gram-negative bacteria plays a key role in the development of endotoxemia, affecting the animal’s immune system. The persistence of endotoxemia can induce weakness and eventual death [13,31].

## 5. Conclusions

In conclusion, abomasal sand impaction contributed to severe obstruction of the abomasum (true stomach), impeded the normal passage of ingesta, and exposed the stomach contents to the mucosa. This resulted in reduced nutrient absorption, electrolytic imbalances, weight loss, and a compromised immune system. Mechanical damage or excoriation likely ensued from the abrasive action of sand against the stomach’s mucosa and submucosa, resulting in erosions and ulceration of the mucosa and these defects likely facilitated the invasion of microbes, potentially leading to endotoxemia and shock. The impacted stomach exerted physical pressure on surrounding organs, including the diaphragm and the respiratory system, potentially disrupting homeostasis, including splanchnic perfusion and pulmonary aeration. This may have contributed to lung lesions and infection.

## Data Availability

Data are contained within the article.
